# Clinical application of dual-phase F-18 sodium-fluoride bone PET/CT for diagnosing surgical site infection following orthopedic surgery

**DOI:** 10.1097/MD.0000000000014770

**Published:** 2019-03-15

**Authors:** Jeong Won Lee, Shi Nae Yu, Ik Dong Yoo, Min Hyok Jeon, Chang-Hwa Hong, Jai-Joon Shim, Sung-Hae Chang, Sang Mi Lee

**Affiliations:** a Department of Nuclear Medicine, International St. Mary's Hospital, Catholic Kwandong University College of Medicine, Seo-gu, Incheon; b Department of Internal Medicine; c Department of Nuclear Medicine; d Department of Orthopedic Surgery; e Department of Neurosurgery, Soonchunhyang University Cheonan Hospital, Dongnam-gu, Cheonan, Chungcheongnam-do, Korea.

**Keywords:** F-18 sodium fluoride, orthopedic surgery, positron emission tomography, surgical site infection

## Abstract

F-18 sodium-fluoride (NaF) bone positron emission tomography (PET/CT) has been used for diagnosing various bone and joint diseases, and, with using dual-phase scan protocol, it could give the same information obtained by the 3-phase bone scintigraphy. The present study aimed to evaluate the diagnostic ability of dual-phase F-18 NaF bone PET/CT in detecting surgical site infection after orthopedic surgery.

Twenty-three patients who underwent dual-phase F-18 NaF bone PET/CT under clinical suspicion of surgical site infection of the bone following orthopedic surgery were enrolled in this study. Dual-phase bone PET/CT consisted of an early phase scan performed immediately after radiotracer injection and a conventional bone-phase scan. All dual-phase PET/CT images were visually assessed, and, for quantitative analysis, 6 parameters of dual-phase PET/CT (lesion-to-blood pool uptake ratio, lesion-to-bone uptake ratio, and lesion-to-muscle uptake ratio on both early phase and bone-phase scans) were measured.

Surgical site infection was diagnosed in 14 patients of the 23 patients. The sensitivity, specificity, and accuracy of visual analysis of dual-phase F-18 NaF bone PET/CT for diagnosing surgical site infection of the bone were 92.9%, 100.0%, and 95.7%, respectively. Among the 6 parameters, the lesion-to-blood pool uptake ratio on early phase scan showed the highest area under the receiver operating characteristic curve value (0.857, 95% confidence interval, 0.649–0.966), with the cut-off value of 0.88 showing sensitivity, specificity, and accuracy of 85.7%, 88.9%, and 87.0%, respectively.

Our study showed the high diagnostic ability of dual-phase F-18 NaF bone PET/CT for detecting surgical site infection following orthopedic surgery. Further studies are needed to compare the diagnostic ability of dual-phase bone PET/CT with other imaging modalities.

## 1 Introduction

Surgical site infection, which is defined as microbial contamination of the surgical wound within 30 days after surgery or within 1 year after surgery with an implant, is the one of the most serious complications after orthopedic surgery.^[1,2]^ The incidence of surgical site infection has been reported to be 1.0% to 2.5% for orthopedic surgery.^[2,3]^ Surgical site infection can significantly increase patient morbidity, re-hospitalization rates, the requirement for additional treatment, and the overall cost of treatment, and can result in disability, deformity, and even death.^[2,4,5]^ Therefore, accurate diagnosis as well as appropriate preventive measures for the infection is crucial to reduce the morbidity, mortality, and economic burden caused by surgical site infection, and various imaging examinations have been used to facilitate the clinical diagnosis of surgical site infection.^[2,6]^ However, detection of surgical site infections after orthopedic surgery may be influenced by the artifacts induced by metallic instrumentation and implants and post-operative inflammation.^[6]^ Therefore, various methods have been studied to enhance the diagnostic ability of imaging examinations.^[7,8]^

Bone positron emission tomography/computed tomography (PET/CT) using F-18 sodium-fluoride (NaF), a highly bone-specific radiotracer, has been widely used for various purposes in bone and joint diseases including detection of bone metastasis, evaluation of joint disorder and rheumatic disease, and assessment of the degree of bone formation.^[9–12]^ A dual-phase F-18 NaF bone PET/CT imaging protocol has been recently introduced to enhance the method's ability to detect inflammatory diseases.^[13]^ In these dual-phase imaging protocols, early-phase PET imaging performed within 10 minutes after F-18 NaF injection is additionally acquired along with the standard bone-phase PET/CT imaging which is performed 30–90 minutes after radiotracer injection.^[13–17]^ Because F-18 NaF is known to show similar pharmacological behavior as diphosphonate bone scanning agents, early-phase F-18 NaF bone PET/CT imaging can provide the same information obtained by the perfusion- and blood-pool-phase images of the 3-phase bone scintigraphy using diphosphonate, indicating that an increased radiotracer uptake on early-phase bone PET/CT images represents regional hyperemia.^[13,16]^ Because inflammatory processes in the tissue are accompanied by regional hyperemia, recent studies have revealed the clinical usefulness of dual-phase F-18 NaF bone PET/CT imaging for detecting inflammatory bone and joint lesions.^[13–16,18]^ We have also reported 2 case reports in which dual-phase bone PET/CT successfully detected inflammatory foci in patients who had undergone spinal surgery.^[14,18]^

Based on our published case reports, we now routinely perform dual-phase F-18 NaF bone PET/CT for patients with clinically suspected infectious bone and joint inflammation at our medical center. In the present study, we retrospectively reviewed the data of patients who underwent dual-phase F-18 NaF bone PET/CT for the detection of surgical site infection after orthopedic surgery and evaluated the diagnostic ability of dual-phase F-18 NaF bone PET/CT.

## 2 Methods

### 2.1 Patients

We retrospectively reviewed the medical records of all patients who underwent F-18 NaF bone PET/CT between January 2015 and July 2018, and recruited 26 patients who underwent dual-phase F-18 NaF bone PET/CT under clinical suspicion of deep surgical site infection of the bone following orthopedic surgery based on the symptoms and findings of a physical examination, blood tests, or imaging examinations. A total of 23 patients (10 men and 13 women; age range, 27–80 years) were eventually enrolled in the present study. All enrolled patients underwent F-18 NaF bone PET/CT within 1 year after orthopedic surgery (median, 2.0 months; range, 0.7–10.6 months). Patients who had metastatic bone lesion (n = 1) or a history of malignant disease (n = 1) or metabolic bone disease (n = 1) were excluded from the study. Clinical information including the results of laboratory and imaging examinations, final diagnosis, and outcome results of the patients were obtained from the medical records. Surgical site infection of the bone was defined by the presence of positive intraoperative cultures on a revision surgery or a positive sign of infection in a clinical examination or imaging test performed within a clinical follow-up period of 1 year. Patients with surgical site infection were classified into acute and chronic infections according to the duration of infection. The institutional review board of Soonchunhyang University approved this study and the study protocol was in accordance with the ethical standards laid down in the Declaration of Helsinki. Informed consent for dual-phase F-18 NaF bone PET/CT was obtained from all individual patients included in this study.

### 2.2 Dual-phase F-18 NaF bone PET/CT

All F-18 NaF bone PET/CT images were acquired using a hybrid PET/CT scanner (Biograph mCT 128 scanner, Siemens Healthcare, Knoxville, TN). Dual-phase bone PET/CT consisted of an early-phase scan, performed immediately after the F-18 NaF administration, and the conventional bone-phase scan. PET imaging in both scans was performed with static acquisition. No special patient preparation was performed before administration of F-18 NaF. For early-phase bone PET/CT scan, the affected part of the patient was placed at the center of the field of view. Immediately after intravenous injection of 185 MBq (5 mCi) of F-18 NaF, early-phase imaging was performed with an initial noncontrast-enhanced CT scan of 100 mA and 120 kVp and a subsequent PET scan in one or 2 bed positions with a 3-dimensional mode at 2 minutes per bed position. Next, conventional bone-phase PET/CT imaging was performed approximately 45 minutes after the F-18 NaF injection with the same setting used in the early-phase scan. The PET images were reconstructed using an iterative algorithm using point-spread-function modeling and time-of-flight reconstruction with attenuation correction.

### 2.3 Bone PET/CT data analysis

Two nuclear medicine physicians who were blinded to the diagnoses and clinical outcomes retrospectively analyzed all dual-phase F-18 NaF bone PET/CT images. To avoid an effect of CT overcorrection artifacts caused by metabolic implants, both nonattenuation-corrected and attenuation-corrected PET images were visually assessed in all patients.^[18,19]^ First, the early-phase and bone-phase PET/CT images were visually assessed. For early-phase scans, positive uptake of the bone lesion was defined as increased radiotracer uptake which is higher than the uptake of the blood pool in the suspected infected bone lesion, as the nonaffected bone showed a very limited uptake on early-phase bone PET/CT in a previous study.^[13]^ For bone-phase scans, positive uptake of the bone lesion was defined as increased radiotracer uptake in the suspected infected bone lesion that was greater than the uptake of the adjacent normal vertebral body or nonaffected contralateral bone. Only bone lesions that showed positive uptake on both early-phase and bone-phase images were considered as positive lesions on dual-phase bone PET/CT images, indicating active infectious inflammation.

Next, quantitative analysis of both early-phase and bone-phase scan images was performed. A total of 4 volumes of interest (VOIs) were manually drawn on both early-phase and bone-phase PET/CT images. The standardized uptake value (SUV) was used as a descriptive indicator for the uptakes of the VOIs. A spheroid-shaped VOI was drawn for the suspected infected bone lesion and the maximum SUV of the bone lesion was measured. For reference organ uptake measurement, the mean SUVs of the blood pool, nonaffected normal bone, and muscle were measured. Blood pool uptake was measured by drawing VOIs over the descending aorta in patients with thoracic, lumbar, or sacral bone lesions, the carotid artery in those with skull lesions, the femoral artery in those with femoral or knee joint lesions, and the posterior tibial artery in those with tibial or tarsal bone lesions. Normal bone uptake was measured by drawing VOIs over the nonaffected normal vertebral body or contralateral bones. Muscle uptake was measured using VOIs drawn over the paraspinal muscles in patients with skull, thoracic, lumbar, or sacral bone lesions, the medial compartment muscles of the contralateral thigh in patients with femoral and knee joint lesions, and the soleus muscle in those with tibial or tarsal bone lesions. Using the maximum SUV of the bone lesion and the mean SUVs for the blood pool, normal bone, and muscle, 6 quantitative dual-phase F-18 NaF bone PET/CT parameters (lesion-to-blood pool uptake ratio, lesion-to-bone uptake ratio, and lesion-to-muscle uptake ratio on both early-phase and bone-phase scans) were calculated.

### 2.4 Statistical analysis

Patients were categorized into 2 groups on the basis of their outcomes: patients with and without surgical site infection. The sensitivity, specificity, positive predictive value, negative predictive value, and accuracy of visual analysis using dual-phase F-18 NaF bone PET/CT for the diagnosis of surgical site infection were determined. For the quantitative analyses, the differences in the 6 quantitative parameters of dual-phase bone PET/CT were compared between patients with and without surgical site infection by using the Mann–Whitney *U* test. The diagnostic accuracies of those 6 parameters were then assessed on the basis of the area under the receiver operating characteristic (ROC) curve (AUC) values. Detection rates and parameters of bone PET/CT were compared between patients with acute and chronic infections by using the Fisher's exact test and Mann–Whitney *U* test. Statistical analyses were performed using MedCalc Statistical Software version 18.9 (MedCalc Software bvba, Ostend, Belgium). A *P*-value of <.05 was considered to indicate a statistical significance.

## 3 Results

### 3.1 Patient characteristics

**Figure 1:**
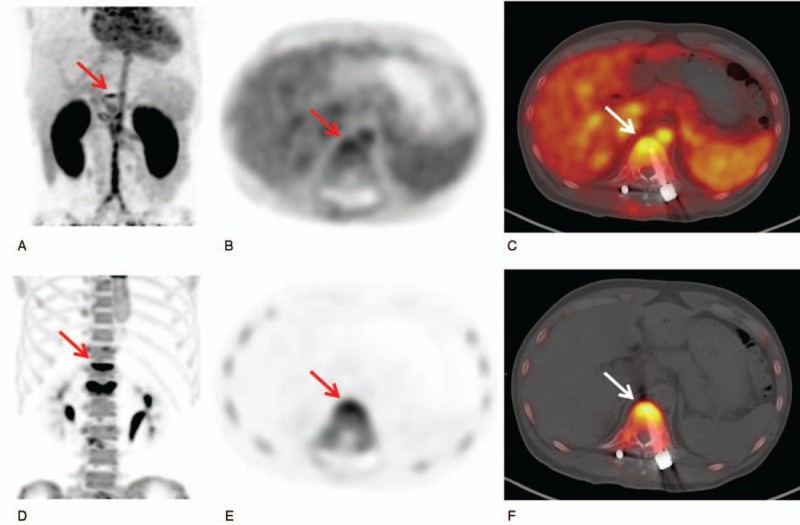
A maximal intensity projection image (A) and transaxial PET (B) and fused PET/CT (C) images from the early-phase scan and a maximal intensity projection image (D) and transaxial PET (E) and fused PET/CT (F) images from the bone-phase scan in F-18 NaF bone PET/CT of a 34-year-old man. Both early-phase and bone-phase scan images showed focally increased radiotracer uptake at the surgical site of the T12 spine (arrows in A–F). The patient underwent a revision operation and was diagnosed with surgical site infection of the spine. PET/CT = positron emission tomography/computed tomography.

**Figure 2:**
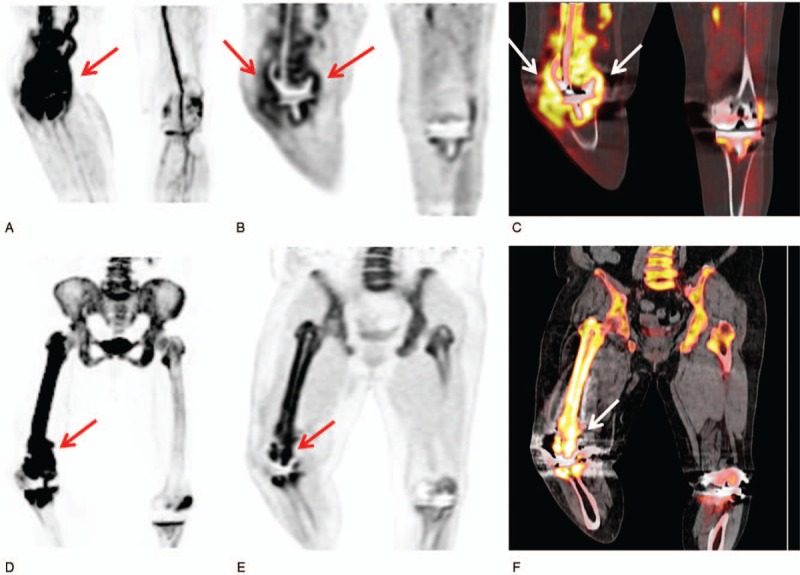
A maximal intensity projection image (A) and coronal PET (B) and fused PET/CT (C) images from the early-phase scan and a maximal intensity projection image (D) and coronal PET (E) and fused PET/CT (F) images from the bone-phase scan in F-18 NaF bone PET/CT of a 68-year-old man. Intensely increased radiotracer uptake around the prosthesis in the right femur and knee joint is shown on both early-phase and bone-phase scan images (arrows in A–F). The patient underwent a revision operation and was diagnosed with surgical site infection of the right femur and knee joint. PET/CT = positron emission tomography/computed tomography.

The clinical characteristics of the 23 enrolled patients are shown in Table 1. The most common site of the suspected infected bone lesion in the enrolled patients was the thoracic and lumbar spine (12 patients; 52.2%; Fig. 1), followed by the femur, knee joint, and tibia (Fig. 2), tarsal bones, skull, and sacrum. On blood tests, 8 patients (34.8%) showed elevated white blood cell counts (normal range, 4.0–10.0 × 10^9^ cells/L) and 11 patients (47.8%) showed elevated serum C-reactive protein level (normal range, 0.0–4.99 md/dL). Only 1 patient showed elevated serum creatinine level of 2.44 mg/dL (normal range, 0.50–1.20 mg/dL). Surgical site infection of the bone was diagnosed in 14 patients (60.9%) of the 23 patients (thoracic and lumbar spine, 6 patients; femur, knee joint, and tibia, 4 patients; tarsal bones, 2 patients; skull, 2 patients). No significant difference of time interval between orthopedic surgery and dual-phase F-18 NaF bone PET/CT was shown between patients with surgical site infection (median, 2.5 months; range, 0.9–10.6 months) and without infection (median, 2.0 months; range, 0.7–7.0 months; *P* = .85). Of 14 patients with surgical site infections, 9 patients (9/14, 64.3%) were diagnosed with acute infection and the remaining 5 patients (5/14, 35.7%) were chronic infection. All 14 patients with surgical site infection underwent a revision surgery, and, among those 14 patients, 5 patients (5/14, 35.7%) were diagnosed with gram-positive infection and 1 patient (1/14, 7.1%) was gram-negative infection on gram staining.

**Table 1:**
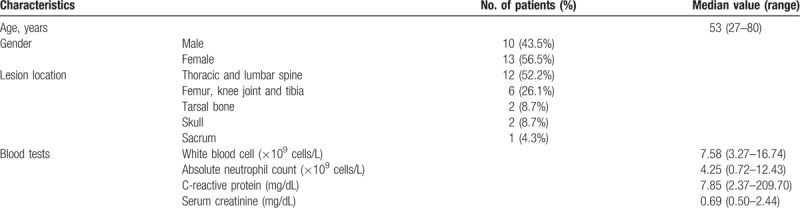
Patient characteristics.

### 3.2 Visual analysis

On visual analysis, all 23 patients showed positive uptake of the bone lesion on bone-phase images of F-18 NaF bone PET/CT. In contrast, on early-phase scan images, 13 patients showed positive uptake of the bone lesion and the remaining 10 patients showed only limited radiotracer uptake uptake in the surgical sites. Thus, 13 patients were determined to have positive lesions on dual-phase F-18 NaF bone PET/CT scans. All 13 patients were diagnosed with surgical site infection (true-positive, 13 patients; false-positive, none). The patient who had elevated serum creatinine level also showed positive uptake on both early-phase and bone-phase images and diagnosed with surgical site infection. All 6 patients with positive results on gram staining showed positive uptake on both early-phase and bone-phase images. Of the remaining 10 patients with no positive lesions on dual-phase bone PET/CT, 1 was diagnosed with surgical site infection (true-negative, 9 patients; false-negative, one patient). One patient with a skull lesion showed negative findings on dual-phase bone PET/CT images, but, was diagnosed with chronic infection of a surgical site. The sensitivity, specificity, positive predictive value, and negative predictive value of visual analysis of dual-phase F-18 NaF bone PET/CT images for diagnosing surgical site infection of the bone were 92.9%, 100.0%, 100.0%, 90.0%, respectively, with an overall accuracy of 95.7%.

### 3.3 Quantitative analysis

**Figure 3:**
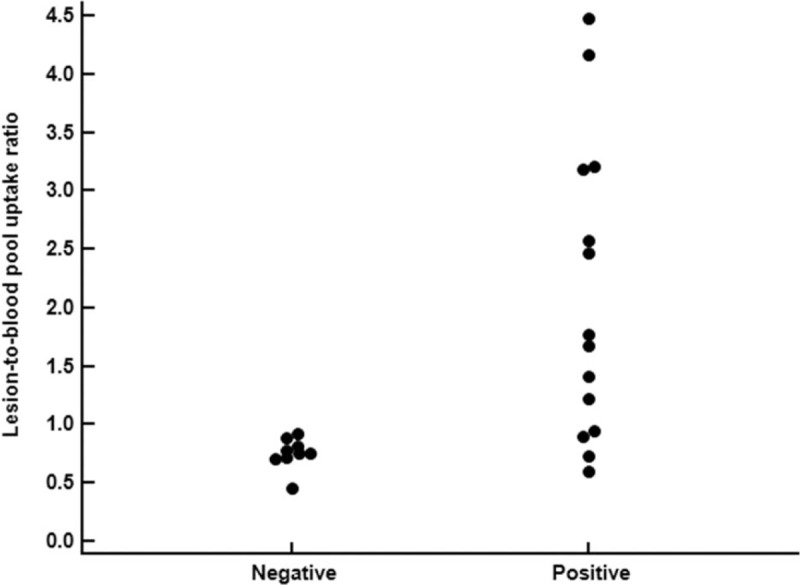
Distribution of the lesion-to-blood pool uptake ratio on early-phase scans in F-18 NaF bone PET/CT in patients with (positive) and without (negative) surgical site infection. PET/CT = positron emission tomography/computed tomography.

Comparisons of the lesion-to-reference organ uptake ratios on early-phase and bone-phase F-18 NaF bone PET/CT between patients with and without surgical site infection are depicted in Table 2. Among the measured parameters, only the lesion-to-blood pool uptake ratio on early-phase images in patients with surgical site infection was significantly higher than that in the other patients (*P* = .01; Fig. 3). In a patient group with surgical site infection, there was no significant difference of the lesion-to-blood pool uptake ratio on early-phase images between 9 patients with acute infection (2.22 ± 1.35) and 5 patients with chronic infection (1.87 ± 1.23; *P* = .62). The patients who had elevated serum creatinine level and diagnosed with surgical site infection also showed increased lesion-to-blood pool uptake ratio of 1.43. The other parameters on early-phase and bone-phase images also showed higher mean values in patients with infections. However, the lesion-to-bone uptake ratio on early-phase images only showed borderline significance (*P* = .08), and the other parameters showed no significant differences between patients with and without infections (*P* > .05).

**Table 2:**
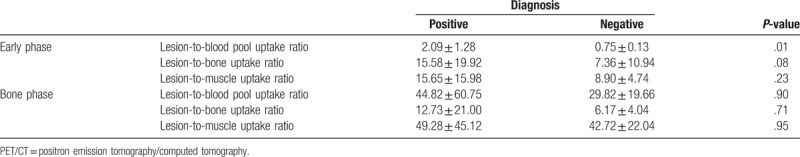
Comparisons of lesion-to-reference organ uptake ratios on early-phase and bone-phase bone PET/CT between patients with (14 patients) and without (9 patients) surgical site infection.

**Figure 4:**
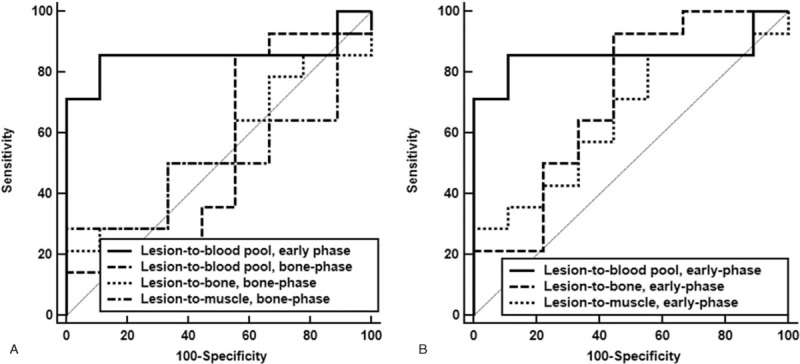
(A) Comparison of ROC curves for the lesion-to-blood pool uptake ratio on the early-phase scan, and the lesion-to-blood pool uptake ratio, lesion-to-bone uptake ratio, and lesion-to-muscle uptake ratio on the bone-phase scan. (B) Comparison of ROC curves for the lesion-to-blood pool uptake ratio, lesion-to-bone uptake ratio, and lesion-to-muscle uptake ratio on the early-phase scan. ROC = receiver operating characteristic.

On ROC curve analysis, the lesion-to-blood pool uptake ratio on early-phase scans had the highest AUC value (0.857, 95% confidence interval, 0.649–0.966; Table 3). For this parameter, the sensitivity, specificity, positive predictive value, negative predictive value, and accuracy with an optimal cut-off value of 0.88 were 85.7%, 88.9%. 92.3%, 80.0%, and 87.0%, respectively. The detection rates of the lesion-to-blood pool uptake ratio for acute infection and chronic infection were 88.9% (8 out of 9 patients) and 80.0% (4 out of 5 patients), respectively (*P* > .99). In pairwise comparisons of AUC values between the lesion-to-blood uptake ratio on early-phase scan and other quantitative parameters, the lesion-to-blood pool uptake ratio on early-phase scans showed significantly higher AUC values than all other parameters of bone-phase scan (*P = *.01 for lesion-to-blood pool uptake ratio, lesion-to-bone uptake ratio, and lesion-to-muscle uptake ratio; Fig. 4A). In comparisons of the AUC values between parameters of early-phase scans, there were no significant differences between the lesion-to-blood pool uptake ratio and the lesion-to-bone uptake ratio (*P = *.20) and between the lesion-to-blood pool uptake ratio and the lesion-to-muscle uptake ratio (*P = *.06; Fig. 4B).

**Table 3:**
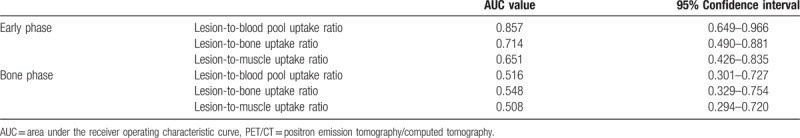
The area under the receiver operating characteristic curves (AUC) values of parameters of dual-phase F-18 NaF bone PET/CT.

## 4 Discussion

In the present study, we demonstrated the clinical utility of dual F-18 NaF bone PET/CT for detecting surgical site infection after orthopedic surgery. Visual analysis of the dual bone PET/CT images showed positive findings in 13 out of 14 patients with surgical site infection, with a sensitivity of 92.9%, specificity of 100.0%, and accuracy of 95.7%. Furthermore, in quantitative analyses, patients with surgical site infections showed significantly higher lesion-to-blood pool uptake ratios on early-phase scans than those without infections, and the lesion-to-blood pool uptake ratio on early-phase scans was considered to be the most suitable parameter for differentiating surgical site infections with an AUC value of 0.857.

The first clinical experience of F-18 NaF bone PET/CT with early-phase scan was reported by Freesmeyer et al.^[13]^ In their study, 11 patients with chronic osteomyelitis in the lower extremity underwent dual-phase bone PET/CT consisting of an early-phase dynamic scan during the first 5 minutes after F-18 NaF injection and a standard bone-phase scan. The results of their study revealed significantly increased radiotracer uptake in the affected bone lesion on the early-phase scan, while only a very limited radiotracer uptake was observed in healthy bone structure, suggesting that the early-phase scan in bone PET/CT is comparable to the perfusion- and blood-pool-scan in 3-phase bone scintigraphy. Two subsequent studies assessed the clinical role of dual-phase F-18 NaF bone PET/CT for detecting septic hip prostheses in 45 patients and evaluating inflammatory sacroiliac joint lesions in 13 ankylosing spondylitis patients.^[15,16]^ In those studies, dual-phase bone PET/CT demonstrated great potential in differentiating septic hip prostheses from aseptic loosening and inflammatory lesions from chronic lesions of ankylosing spondylitis. In the present study, based on the findings of regional hyperemia on early-phase bone PET/CT, dual-phase bone PET/CT showed considerable diagnostic ability in differential diagnosis of surgical site infection of orthopedic surgery. Considering the limited uptake of surgical sites on early-phase images in patients without negative infection in our study, simple postoperative changes in the bone do not accompany with increased early-phase F-18 NaF uptake, similar to nonaffected normal bone structure.^[13]^ In contrast to the protocol used by Freesmeyer et al^[13]^, we used a simplified static imaging acquisition protocol for early-phase scans that can be easily performed in a routine clinical setting and this simplified imaging protocol provided sufficient information for the diagnosis. Theoretically, dual-phase bone PET/CT only provides information similar to that acquired with 3-phase bone scintigraphy. However, because of the high spatial resolution and 3-dimensional image acquisition in PET scans, dual-phase bone PET/CT can be used for suspected orthopedic infections in which the use of 3-phase bone scan is limited, such as spinal infections.^[14,20]^

In addition to visual analysis, we also measured quantitative indices to assess their clinical utility. Because the clinical value of SUV measured on F-18 NaF bone PET has not been defined,^[10,17]^ we used the lesion-to-reference organ uptake ratios on both early-phase and bone-phase bone PET/CT scans, which were not assessed in the previous studies.^[13,15,16]^ On bone-phase scan in bone PET/CT, increased lesion-to-reference organ uptake ratios were observed at the surgical sites irrespective of infection, similar to the bone scintigraphy findings after orthopedic surgery.^[21,22]^ Therefore, the quantitative parameters of bone-phase scans showed low diagnostic ability for detecting surgical site infection with AUC values between 0.508 and 0.548. On the other hand, the lesion-to-blood pool uptake ratio on the early-phase scan showed significant differences between patients with and without infection and the highest AUC value of 0.857 among the indices. The results of visual and quantitative analyses in our study indicated that surgical site lesions that showed increased radiotracer uptake which is higher than the activity of the artery on early-phase bone PET/CT images should be considered as infectious conditions.

For decades, various radionuclide imaging methods have been used for detecting bone and joint infections. Three-phase planar bone scintigraphy using Tc-99m-labeled diaphosphonate is the most widely available nuclear medicine imaging examination and has shown high sensitivity for detecting osteomyelitis.^[20,22]^ However, it has low specificity and accuracy, and its 2-dimensional nature limits its effectiveness for evaluation of infected lesions located behind the great vessel or heart.^[14,20,23]^ Recently, clinical studies with Tc-99m-labeled diaphosphonate bone scintigraphy using single photon emission computed tomography (SPECT)/CT have shown encouraging results in overcoming those limitations of bone scintigraphy, demonstrating high accuracy for detecting diabetic foot osteomyelitis, periprosthetic infection in knee joints, and skull base osteomyelitis.^[24–26]^ As both F-18 NaF and Tc-99m-labeled diaphosphonate are bone-seeking radiotracers with having similar pharmacological kinetics to each other,^[13]^ further clinical study would be needed to compare the diagnostic ability between Tc-99m-labeled diaphosphonate SPECT/CT and F-18 NaF bone PET/CT. In vitro labeled leukocyte scintigraphy using In-111 or Tc-99m has shown high accuracy for prosthetic infection.^[22]^ However, because leukocytes normally accumulate in bone marrow as well as infected bone, additional bone marrow imaging with Tc-99m sulfur colloid is needed to differentiate osteomyelitis from normal marrow uptake.^[22]^ Moreover, decreased leukocyte uptake is frequently encountered in spinal infection, especially for gram-negative vertebral infection, with the accuracy being 66% for detecting vertebral osteomyelitis.^[27,28]^ Ga-67 scintigraphy has high diagnostic ability for vertebral osteomyelitis, and is known as the primary gamma camera imaging method for vertebral osteomyelitis.^[20,22]^ Nevertheless, it is not an optimal radionuclide for gamma camera imaging and the relatively long interval between radiotracer injection and imaging precludes the wide use of Ga-67 scintigraphy.^[29]^ Considering the results of our study, dual-phase F-18 NaF bone PET/CT can be an alternative nuclear medicine imaging modality for detecting orthopedic infection, especially spinal infection, and can overcome the limitations of the aforementioned radionuclide imaging methods. Several recent studies have evaluated the clinical role of F-18 fluorodeoxyglucose (FDG) PET/CT for diagnosing orthopedic infection and showed that FDG PET/CT has high diagnostic ability that outperforms conventional radionuclide imaging modalities.^[8,22,30–32]^ However, delayed union of the bone and foreign body reaction of the orthopedic implants could also show increased FDG uptake even in the absence of infection, causing false-positive findings.^[31,32]^ Conversely, our previous case report described a patient with surgical site infection of the spine that was detected by dual-phase F-18 NaF bone PET/CT, but only showed false-negative finding on FDG PET/CT.^[14]^ Because dual-phase F-18 NaF bone PET/CT and FDG PET/CT use different mechanisms to show uptake for the infected lesion,^[8,13,22]^ both imaging methods could have complementary roles in the diagnosis of infection and a comparative study between the 2 methods is needed.

Clinical conditions of patients such as renal function and duration of infection can affect the image quality and diagnostic ability of Tc-99m-labeled diaphosphonate bone scintigraphy for detecting bone infection.^[33,34]^ Although F-18 NaF bone PET/CT has better image resolution with a high bone-to-background uptake ratio than conventional bone scintigraphy,^[19]^ the impact of those clinical conditions might be also considered as a problem in F-18 NaF bone PET/CT. However, almost all of injected F-18 NaF is known to be retained by bone after a single pass of blood and previous study has found that the image quality of F-18 NaF bone PET/CT in patients with end stage renal disease was not inferior to that in those with normal renal function.^[35,36]^ Furthermore, the first clinical study with dual-phase bone PET/CT has already shown increased uptake ratio between diseased and healthy contralateral bones in patients with chronic osteomyelitis.^[13]^ In our study, dual-phase F-18 NaF bone PET/CT successfully detected surgical site infection in a patient with elevated serum creatinine level. Nevertheless, although there was no statistical significance, the patients with chronic infection showed lower values of the lesion-to-blood pool uptake ratio on early-phase images. Moreover, the patient with false-negative finding on visual analysis was also diagnosed with chronic infection. Therefore, the duration of the infection might affect the results of the early-phase bone PET/CT, but, considering the small number of the patients in the present study, further studies are warranted. In addition, to gain wider acceptance for using F-18 NaF bone PET/CT, the diagnostic accuracy of dual-phase bone PET/CT should be compared between gram-positive and gram-negative infections in further studies.

The study had several unaddressed limitations. First, the study was performed with only a small number of patients and the results require further validation. Because we are continuing to perform dual-phase F-18 NaF bone PET/CT for patients with suspected bone and joint infection at our medical center, we aim to carry out a further study with more patients in the future. Second, because of the small number of enrolled patients with diverse clinical conditions, comparison of the diagnostic ability with that of other imaging examinations was not performed in the present study. Our future study will be able to deal with this subject. Third, although we also reviewed nonattenuation-corrected PET images in the visual analysis, increased radiotracer activity due to CT overcorrection artifacts caused by orthopedic hardware might affect the results of visual analysis^[18,19,37]^ Finally, not all of the enrolled patients had undergone surgical exploration. Consequently, histopathological confirmation was not available in some of the patients, mainly those with negative diagnosis.

In conclusion, the results of our study demonstrated that, with using lesion-to-blood pool uptake ratio on early-phase scan, dual-phase F-18 NaF bone PET/CT could be a promising imaging modality for diagnosing surgical site infection after orthopedic surgery. Both visual analysis and quantitative analysis using lesion-to-blood pool uptake ratio on early-phase scan showed high sensitivity and specificity for the discrimination of surgical site infection. To validate the results of the study, larger studies performing comparative analysis with other imaging modalities should be executed.

## Author contributions

**Conceptualization:** Jeong Won Lee, Sang Mi Lee.

**Data curation:** Shi Nae Yu, Min Hyok Jeon, Chang-Hwa Hong, Jai-Joon Shim, Sung-Hae Chang, Sang Mi Lee.

**Formal analysis:** Jeong Won Lee, Ik Dong Yoo, Sung-Hae Chang, Sang Mi Lee.

**Funding acquisition:** Jeong Won Lee, Sang Mi Lee.

**Investigation:** Ik Dong Yoo, Sang Mi Lee.

**Methodology:** Jeong Won Lee, Shi Nae Yu, Min Hyok Jeon, Chang-Hwa Hong, Jai-Joon Shim, Sang Mi Lee.

**Supervision:** Sang Mi Lee.

**Writing – original draft:** Jeong Won Lee, Ik Dong Yoo.

**Writing – review & editing:** Shi Nae Yu, Ik Dong Yoo, Min Hyok Jeon, Chang-Hwa Hong, Jai-Joon Shim, Sung-Hae Chang, Sang Mi Lee.

Sang Mi Lee orcid: 0000-0002-7943-3807.
